# Willingness to Try Pelvic Floor Physiotherapy Among Women Attending Gynecology Clinics in Saudi Arabia: A Cross-Sectional Study of Beliefs, Barriers, and Facilitators

**DOI:** 10.5152/tud.2026.26048

**Published:** 2026-10-16

**Authors:** Hanan Mohammed Shamrani

**Affiliations:** Department of Obstetrics and Gynecology, King Abdul Aziz University, Jeddah, Kingdom of Saudi Arabia

**Keywords:** Barriers, beliefs, pelvic floor physiotherapy, pelvic organ prolapse, urinary incontinence, willingness

## Abstract

**Objective:**

Pelvic floor physiotherapy (PFPT) is a first-line conservative treatment for pelvic floor dysfunction. This study assessed the beliefs, attitudes, perceived barriers, and willingness of women attending obstetrics and gynecology (OBGYN) clinics in Jeddah, Saudi Arabia, to try PFPT.

**Methods:**

A cross-sectional study was conducted among 200 women attending OBGYN outpatient clinics at King Abdulaziz University Hospital, Jeddah, Saudi Arabia, between May 10, 2025, and August 30, 2026. Participants completed a bilingual questionnaire assessing demographics, medical and obstetric history, knowledge, beliefs, perceived barriers, and willingness to try PFPT. Associations were analyzed using chi-square, Fisher’s exact, and independent *t* tests.

**Results:**

In the current study, 200 participants were included. Willingness to try PFPT was 64.0% if offered free and increased to 84.0% when recommended by a physician; 91.5% agreed or strongly agreed they would try PFPT if advised by their doctor. Knowledge was generally good, with 65.5% recognizing that pelvic floor exercises reduce urinary leakage and 87.5% expressing confidence in learning them. Financial cost was the most reported barrier (56.5%), followed by difficulty accessing services (48.0%). The preferred delivery mode was in-person care with a female physiotherapist (72.5%), followed by video- or app-based home programs (39.5%). Although 24% reported urinary incontinence, only 11% had previously consulted a physiotherapist.

**Conclusion:**

Women attending OBGYN clinics showed high willingness to try PFPT, particularly when recommended by a physician. Addressing cost and access barriers, increasing physician endorsement, ensuring availability of female physiotherapists, and expanding digital delivery options may improve PFPT uptake.

**Main Points**

- Nearly two-thirds (64.0%) of participants were willing to try pelvic floor physiotherapy (PFPT) if offered free of charge, increasing to 84.0% when PFPT was recommended by a physician.
- Participants generally demonstrated favorable knowledge and attitudes toward PFPT, with 87.5% being confident that they could learn pelvic floor exercises if appropriately instructed.
- Financial cost and difficulty accessing physiotherapy services were the most prominent perceived barriers.
- Despite urinary incontinence being reported by 24.0% of participants, only 11.0% had previously consulted a physiotherapist for a pelvic floor problem.
- Physician recommendation, affordable access, female physiotherapists, and flexible delivery options may represent important targets for improving PFPT uptake.

## Introduction

Pelvic floor disorders, such as urinary incontinence (UI), pelvic organ prolapse (POP), and fecal incontinence, are a significant health burden that affects 1 in 4 females during their lifetime.^1^ Its prevalence is up to 46% among adult females and significantly higher with higher age, parity, and obesity.^2^ UI alone is experienced by more than 500 million people around the world, and by the age of 85 years, about 1 in 5 women will have had prolapse surgery or incontinence surgery. These conditions have a significant negative impact on quality of life, sexual function, and social participation and place a massive burden on health services.^2,3^ The American College of Obstetricians and Gynecologists (ACOG) recommends conservative treatment of stress, urgency, and mixed UI by pelvic floor muscle training (PFMT) as first-line treatment.^4^ A review^5^ found that 74% of women who underwent PFMT reported cure or improvement, compared with 11% of women who received no treatment. International Urogynecology Consultation^6^ provides high-level Randomized controlled trials (RCTs) evidence supporting the use of PFMT as a first-line treatment for POP and showing that symptom severity was reduced with PFMT. Pelvic floor physiotherapy (PFPT) has thus been characterized as having robust evidence-based support for most pelvic floor disorders.^7^ However, the evidence base for PFPT is far from being widely used, especially in the Gulf region. The prevalence of UI among women in the Gulf countries is between 20.3% and 54.5%, but many of them do not seek medical advice.^8^ In Saudi Arabia, specifically, the prevalence of UI has been reported to be between 30% and 44.2%, with health-seeking behavior being always poor.^9-11^ A study reported that the general knowledge of the Saudi women in relation to UI is satisfactory, but the specific knowledge is very low, and they had never considered it as a medical condition even though they were embarrassed about treatment, particularly from male physicians, with 16.8% of them saying that they simply did not consider UI as a medical condition.^9^ There are additional factors to consider when using PFPT in Saudi Arabia that are sociocultural. Beliefs about modesty, cultural values and norms, and the desire for same-sex healthcare providers could affect women’s willingness to seek out pelvic floor care.^12,13^ In addition, the family has a strong influence on health service utilization in collectivist societies in the Gulf, which can be a barrier or a facilitator to health service utilization.^14^ Although these culturally specific factors exist, no study has yet focused specifically on the Saudi women’s willingness to try PFPT, and the barriers that might prevent them from using it. Therefore, this is the first study to address these interconnected domains in this context. We aimed to assess the beliefs, attitudes, and willingness of females attending obstetrics and gynecology (OBGYN) clinics in Jeddah, Saudi Arabia, to try PFPT, including the role of physician recommendation in influencing willingness, to provide evidence-based insights for clinicians and policymakers seeking to enhance patient education, referral practices, and culturally appropriate service delivery.

## Material and Methods

### Study Design and Settings

A cross-sectional study was conducted among females attending OBGYN outpatient clinics at King Abdulaziz University Hospital, Jeddah, Saudi Arabia. The study was conducted between May 10, 2025, and August 30, 2026. The study was performed in accordance with the Strengthening the Reporting of Observational Studies in Epidemiology (STROBE) guidelines for cross-sectional studies. No artificial intelligence or large language model (LLM) tools were used during the conduct of the study or the preparation of this manuscript.

### Population and Eligibility Criteria

The target population comprised females attending OBGYN clinics in Jeddah, either as patients or visitors. Eligible participants were females aged 18 years or older, able to read and understand Arabic or English, and willing to provide informed consent. Males and females under 18 years of age were excluded. Participants were recruited through convenience sampling by approaching eligible females in the clinic waiting areas and inviting them to participate.

### Data Collection

Data were collected using a structured, self-administered questionnaire distributed via a Google Forms link. The questionnaire was available in both Arabic and English and required approximately 10-15 minutes to complete. The instrument comprised 8 sections: (1) demographic characteristics, including age, educational level, marital status, employment status, and region of residence; (2) obstetric and medical history, including parity, mode of delivery (vaginal, instrumental, and cesarean), history of POP, UI, and previous pelvic surgery; (3) risk factors associated with pelvic floor disorders, including constipation, obesity, heavy lifting, menopause, chronic cough, and amenorrhea (multiple responses allowed); (4) previous experience with PFPT, including prior consultation with a physiotherapist, location of consultation, previous trial of pelvic floor exercises, compliance with sessions, duration of attendance, and perceived improvement; (5) knowledge regarding pelvic floor disorders and physiotherapy, assessed using 4 true/false/unsure items addressing the role of pelvic floor exercises in reducing urinary leakage, the effectiveness of physiotherapy for POP, whether surgery is the only treatment for UI, and whether only postmenopausal women experience pelvic floor disorders; (6) beliefs and attitudes toward PFPT, assessed using 7 items on a 5-point Likert scale (strongly disagree to strongly agree) covering perceived prevalence, treatment effectiveness, self-efficacy, social stigma, prioritization, safety concerns, and willingness if recommended by a physician; (7) perceived barriers to utilizing PFPT, assessed using 7 items on a 5-point scale (not a barrier at all to major barrier) addressing time, cost, access, embarrassment, modesty concerns, lack of belief in effectiveness, and difficulty performing exercises at home without supervision; and (8) willingness and intention to try PFPT, including likelihood of participation if offered free of charge and if recommended by a physician (5-point scale from very unlikely to very likely), preferred delivery methods (multiple responses allowed: in-person clinic with a female physiotherapist, home-based with printed instructions, or home-based with video/app guidance), main reason for unwillingness (single best answer), and the influence of family support on willingness.

The primary end point was willingness to try PFPT, defined as a binary outcome (willing vs not willing). Participants who reported being “likely” or “very likely” to try PFPT were classified as willing. In contrast, those who were “unsure,” “unlikely,” or “very unlikely” were classified as not willing. Willingness was assessed under 2 conditions: if physiotherapy services were provided free of charge and if physiotherapy was recommended by a physician. Secondary end points included awareness and knowledge level about PFPT, beliefs and attitudes toward pelvic floor therapy, perceived barriers to seeking PFPT, and associations between demographic variables and willingness. Regular data quality checks were performed to ensure accuracy, completeness, and consistency of responses. Incomplete or duplicate responses were identified and handled according to prespecified criteria; incomplete responses were excluded from analysis.

### Sample Size and Ethical Considerations

The target sample size was 200 participants. This study was approved by Unit of Biomedical Ethics, Research Ethics Committee, King Abdulaziz University, with reference number 517-25 on March 2, 2025. This study was conducted in accordance with the Declaration of Helsinki and the Saudi Arabian National Committee of Bioethics guidelines. Electronic informed consent was obtained at the beginning of the online questionnaire; participants were required to read and agree to the consent statement before proceeding. Participation was voluntary and anonymous, and participants could withdraw at any time by closing the questionnaire without consequence. No identifying information (name, national identification number, phone number, or email address) was collected. All data were stored in password-protected electronic files accessible only to the principal investigator and retained for a minimum of 5 years after study completion, after which they will be securely deleted. No financial compensation was provided to participants.

### Statistical Analysis

Data were collected, coded, revised, and entered to the R statistical software (RStudio), version 4.4.0 (R project for statistical computing; Vienna, Austria). The data are presented as numbers and percentages for qualitative data; means, standard deviations, and ranges for quantitative data with parametric distribution; and median with interquartile range (IQR) for quantitative data with non-parametric distribution. The Shapiro test was used to verify the normality of distribution.

The chi-square test was used in the comparison between 2 groups with qualitative data, and Fisher’s exact test was used instead of the chi-square test when the expected count in any cell was less than 5.

An independent *t* test was used in the comparison between 2 groups with quantitative data and parametric distribution, and the Wilcoxon–Mann–Whitney test was used in the comparison between 2 groups with quantitative data and non-parametric distribution.

The confidence interval was set to 95%, and the margin of error accepted was set to 5%. So, the *P*-value was considered significant as follows: *P* > .05: non-significant (NS) and *P* < .05: significant (S). In addition to descriptive analyses, inferential analyses were performed to explore factors associated with willingness to try PFPT. Participants were classified as willing if they reported being “likely” or “very likely” to participate and as not willing if they reported being “unsure,” “unlikely,” or “very unlikely.” Demographic characteristics were compared between willing and not willing participants under both scenarios: PFPT offered free of charge and PFPT recommended by a physician.

## Results

### Demographic and Background Information

We included 200 women in the current study. The participants’ age ranged from 10 to 71 years, with a mean ± SD of 36.12 ± 11.32 years and a median age of 36 years. Most participants had a bachelor’s degree (60.5%, 121 participants), followed by secondary education (16%, 16 participants) and postgraduate education (12.5%, 25 participants). While 4.5% (9 participants) had less than secondary education, 6.5% (13 participants) held a diploma.

Regarding marital status, most participants were married (66%, 132 participants), 26.5% (53 participants) were single, 6.5% (13 participants) were divorced, and 1% (2 participants) were widowed. Regarding employment status, participants were employed and housewives (39% and 36.5% respectively), while 17% (34 participants) were students, as shown in Table 1.

**Table 1.** Demographic Characteristics of Participants (N = 200)

Demographic Characteristics	N = 200
Age in Years	
Min.-Max.	18.00-71.00
Mean ± SD	36.12 ± 11.32
Median (IQR)	36.00 (27.00, 43.00)
Educational Level	
Bachelor’s	121.00 (60.50%)
Diploma	13.00 (6.50%)
Less than secondary	9.00 (4.50%)
Postgraduate studies	25.00 (12.50%)
Secondary	32.00 (16.00%)
Marital Status	
Divorced	13.00 (6.50%)
Married	132.00 (66.00%)
Single	53.00 (26.50%)
Widowed	2.00 (1.00%)
Job Level	
Employee	78.00 (39.00%)
Housewife	73.00 (36.50%)
Others	15.00 (7.50%)
Student	34.00 (17.00%)

IQR, interquartile range; Min., minimum; Max., maximum; SD, standard deviation.

### Medical and Obstetric History

Regarding medical and obstetric history, participants had a mean number of 2.06 ± 2.1 children, with a median of 2 children and a range of 0-9 children. The mean number of vaginal births was 1.46 ± 1.92, with a range of 0-9 births. Instrument-assisted deliveries were uncommon, with a mean of 0.2 ± 0.66. The mean number of cesarean births was 0.64 ± 1.15, with a range of 0-6.

Regarding pelvic floor–related conditions, 12% of participants reported a previous diagnosis of POP, while 82.5% reported no previous diagnosis and 5.5% were unsure. A history of UI was reported by 24% of participants, while 68% reported no history of UI and 8% were unsure. Previous pelvic surgery was reported by 10% of participants, while 90% had not undergone any pelvic surgery as shown in Table 2.

**Table 2.** Obstetric and Medical History of Participants (N = 200)

Characteristics	N = 200
Number of Children	
Min.-Max.	0.00-9.00
Mean ± SD	2.06 ± 2.10
Median (IQR)	2.00 (0.00, 4.00)
Number of Vaginal Births	
Min.-Max.	0.00-9.00
Mean ± SD	1.46 ± 1.92
Median (IQR)	0.00 (0.00, 3.00)
Number of Instrument-Assisted Births	
Min.-Max.	0.00-4.00
Mean ± SD	0.20 ± 0.66
Median (IQR)	0.00 (0.00, 0.00)
Number of Cesarean Births	
Min.-Max.	0.00-6.00
Mean ± SD	0.64 ± 1.15
Median (IQR)	0.00 (0.00, 1.00)
Previous Diagnosis of Pelvic Organ Prolapse	
Unsure	11.00 (5.50%)
No	165.00 (82.50%)
Yes	24.00 (12.00%)
History of Urinary Incontinence	
Unsure	16.00 (8.00%)
No	136.00 (68.00%)
Yes	48.00 (24.00%)
Previous Pelvic Surgery	
Previous pelvic surgery	20 (10.0%)
No previous pelvic surgery	180 (90.0%)

IQR, interquartile range; Min., minimum; Max., maximum; SD, standard deviation.

Regarding risk factors associated with pelvic floor disorders, constipation was the most common reported condition, affecting 29.5% of participants, followed by obesity (19.5%), heavy lifting at work (10%), and chronic cough (7.5%). Menopause was reported by 7% of participants, while amenorrhea was reported by only 2%, as shown in Table 3.

**Table 3.** History of Pelvic Floor Disorder Risk Factors (Multiple Responses Allowed) (N = 200)

Risk Factor	n (%)
Constipation	59 (29.5%)
Obesity	39 (19.5%)
Heavy lifting at work	20 (10.0%)
Chronic cough	15 (7.5%)
Menopause	14 (7.00%)
Amenorrhea	4 (2.0%)
None	101 (50.5%)

Note: Participants could select more than one risk factor.

Regarding previous experience with PFPT, 11% of participants had previously consulted a physiotherapist for pelvic floor problems, while 89% had never consulted a physiotherapist for these conditions.

Regarding prior experience with pelvic floor interventions, 22% reported having previously tried pelvic floor exercises or physiotherapy, while 78% had never attempted either intervention. Concerning treatment outcomes, 14% of participants reported experiencing improvement following therapy, 6.5% reported no improvement, and 27% were unsure whether any improvement had occurred, as shown in Table 4.

**Table 4.** Previous Experience with Pelvic Floor Physiotherapy (N = 200)

Items	N = 200
Previously Consulted a Physiotherapist for Pelvic Floor Problems	
Previously consulted a physiotherapist for pelvic floor problems	22 (11.0%)
Never consulted a physiotherapist	178 (89.0%)
Previously Tried Pelvic Floor Exercises or Physiotherapy	
Previously tried pelvic floor exercises or physiotherapy	44 (22.0%)
Never tried pelvic floor exercises or physiotherapy	156 (78.0%)
Improvement After Therapy	
Reported improvement after therapy	28 (14.0%)
No improvement	13 (6.5%)
Unsure about improvement	54 (27.0%)
No answer	105 (52.5%)

### Knowledge

Regarding the knowledge of participants, participants generally demonstrated good knowledge regarding pelvic floor disorders and their management. 65.5% recognized that pelvic floor exercises may help reduce urinary leakage, while 66% acknowledged that physical therapy can improve symptoms of POP. Regarding treatment options for UI, 6.5% agreed with the statement that surgery is the only effective treatment. Similarly, 6% agreed with the statement that only postmenopausal women experience pelvic floor disorders, as shown in Table 5.

**Table 5.** Knowledge Regarding Pelvic Floor Disorders and Physiotherapy (N = 200)

Items	Yes, n (%)	No, n (%)	Unsure, n (%)
Pelvic floor exercises may reduce urinary leakage	131.00 (65.50%)	8.00 (4.00%)	61.00 (30.50%)
Physical therapy can improve prolapse symptoms	132.00 (66.00%)	7.00 (3.50%)	61.00 (30.50%)
Surgery is the only effective treatment for urinary incontinence	13.00 (6.50%)	93.00 (46.50%)	94.00 (47.00%)
Only postmenopausal women experience pelvic floor disorders	12.00 (6.00%)	96.00 (48.00%)	92.00 (46.00%)

### Beliefs and Attitudes

Regarding beliefs and attitudes, participants demonstrated highly positive attitudes toward PFPT. 46.5% agreed and an additional 41% strongly agreed that they would be able to learn and correctly perform pelvic floor exercises if appropriately instructed. Similarly, 54.5% agreed and 19.5% strongly agreed that PFPT is effective in reducing symptoms of UI or POP.

Regarding perceptions of pelvic floor disorders, 20% agreed and 12.5% strongly agreed that pelvic floor problems are common among women of their age group.

Of the participants 15.5 % agreed and 9% strongly agreed that they worried about being judged by others if they sought help for pelvic floor problems.

Most participants did not consider PFPT to be a lower priority than other health needs, as 44% disagreed and 14.4% strongly disagreed with this statement. Likewise, 38% disagreed and 6% strongly disagreed that PFPT could be harmful. All participants expressed willingness to try PFPT if recommended by their physician, with 48.5% agreeing and 43% strongly agreeing with this statement, as shown in Table 6.

**Table 6.** Beliefs and Attitude Toward Pelvic Floor Physiotherapy Among Participants (N = 200)

Items	Strongly Disagree, n (%)	Disagree, n (%)	Neutral, n (%)	Agree, n (%)	Strongly Agree, n (%)
Pelvic floor problems are common among women of my age	9.00 (4.50%)	38.00 (19.00%)	88.00 (44.00%)	40.00 (20.00%)	25.00 (12.50%)
Pelvic floor physiotherapy is effective in reducing symptoms of incontinence or prolapse	2.00 (1.00%)	4.00 (2.00%)	46.00 (23.00%)	109.00 (54.50%)	39.00 (19.50%)
I am confident that I can learn and perform pelvic floor exercises correctly if taught properly	1.00 (0.50%)	2.00 (1.00%)	22.00 (11.00%)	93.00 (46.50%)	82.00 (41.00%)
I worry about how others will judge me if I seek help for pelvic floor problems	18.00 (9.00%)	90.00 (45.00%)	53.00 (26.50%)	31.00 (15.50%)	18.00 (9.00%)
Pelvic floor physiotherapy is a lower priority compared with other health needs	29.00 (14.50%)	88.00 (44.00%)	49.00 (24.50%)	23.00 (11.50%)	11.00 (5.50%)
I am concerned about the safety or potential harm of pelvic floor physiotherapy	12.00 (6.00%)	76.00 (38.00%)	74.00 (37.00%)	32.00 (16.00%)	6.00 (3.00%)
If my doctor recommends pelvic floor physiotherapy, I would be willing to try it	1.00 (0.50%)	3.00 (1.50%)	13.00 (6.50%)	97.00 (48.50%)	86.00 (43.00%)

### Perceived Barriers

Regarding the perceived barriers to utilizing PFPT, financial cost was the most commonly reported obstacle to participating in PFPT, with 21.5% and 35% of participants rating it as a medium and major barrier, respectively. Difficulty obtaining appointments or accessing physiotherapy services was the second most frequently reported barrier, being identified as a medium obstacle by 30% and a major obstacle by 18% of participants.

Lack of sufficient time to attend physiotherapy sessions was reported as a medium (21.5%) or major barrier (4.5%) by participants, while religious or modesty concerns regarding pelvic or internal examinations were perceived as a medium barrier by 13.5% and a major barrier by 7.5%. Difficulty performing exercises at home without supervision was reported as a medium barrier by 15.5% and a major obstacle by 10.5% of participants. Embarrassment about discussing or practicing pelvic floor issues and lack of belief in the benefits of exercise were reported barriers, with only 8% and 3% of participants, respectively, rating them as medium or major obstacles, as shown in Supplementary Table 1.

### Willingness and Intention to Try Physiotherapy

Participants demonstrated a generally favorable willingness to engage in PFPT. If physiotherapy services were provided free of charge, 23.5% of participants reported being very likely to participate and 40.5% reported being likely to participate. Willingness increased when physiotherapy was recommended by a physician, with 36.5% of participants reporting that they would be very likely to participate and 47.5% indicating that they would be likely to participate.

Regarding preferred methods of receiving PFPT, attendance at a clinic with a female physiotherapist was the most preferred option (72.5%), followed by a home-based program delivered through video instructions or a mobile application (39.5%). Home programs using printed instructions were the least preferred approach (16%).

Among participants who were not willing to physiotherapy, financial cost was the most reported reason (48.5%), followed by embarrassment (17.5%) and lack of available resources (15.5%). Preference for surgery or medication was reported by 5.5% of participants, while disbelief in the effectiveness of physiotherapy and religious or modesty concerns were reported by 7.5% and 5%, respectively.

Family support appeared to play an important role in treatment acceptance, as 60.5% of participants indicated that support or approval from a family member would increase their likelihood of participating in PFPT, while 31.5% responded “maybe,” as shown in Supplementary Table 2.

### Association Between Willingness and Demographic Characteristics

For the association between willingness and demographic characters, participants who reported being “likely” or “very likely” to try PFPT if recommended by a physician if it was free were classified as willing (n = 128), while those who were unsure, unlikely, or very unlikely were classified as not willing (n = 72).

Participants who were willing were significantly older than those who were not willing, with a mean ± SD of 37 ± 11 years compared with 34 ± 11 years, respectively (*P* = .047). Regarding marital status, married participants were the most frequent category in both groups and were more common among those willing (70%) than those not willing (58%), whereas the proportion of single participants decreased from 39% among those not willing to 20% with significant difference (*P* = .010), as shown in Supplementary Table 3.

For the association between willingness and demographic characteristics, participants who reported being “likely” or “very likely” to try PFPT if recommended by a physician were classified as willing (n = 168), while those who were unsure, unlikely, or very unlikely were classified as not willing (n = 32). No statistically significant associations were observed between willingness to undergo PFPT and any of the examined demographic characteristics, as shown in Supplementary Table 4.

## Discussion

In this cross-sectional study, we investigated participants attending OBGYN clinics at a tertiary center in Jeddah, Saudi Arabia. The results showed that the majority of the 200 women interviewed showed an interest in PFPT. Their willingness increased from 64.00% when services were presented as free to 84.00% when the women were referred to the services by their physician. The costs associated with PFPT were identified as the major barrier women faced, although the women had a good baseline knowledge and a positive attitude towards PFPT services. Although the burden of pelvic floor disorders in this sample was considerable, with 24% reporting a history of UI and 12% reporting a previous diagnosis of POP, reported prior use of PFPT remained low, with only 11% of participants having ever consulted a physiotherapist for pelvic floor problems. Age and marital status were significantly associated with willingness to try PFPT when it was offered free of charge, whereas no demographic factor was associated with willingness when PFPT was recommended by a physician.

The established components of willingness can be examined based on the capability, opportunity, motivation—behavior (COM-B) model, which provides a framework of capability, opportunity, and motivation.^15^ The present research considered capability indicators to be favorable; 87.5% of participants agreed or strongly agreed that they were confident they could learn and correctly perform pelvic floor exercises if properly instructed, 74% agreed or strongly agreed that PFPT is effective in reducing symptoms of UI or POP, and 65.5% recognized that pelvic floor exercises may help reduce urinary leakage. However, opportunity-related factors, chiefly cost and access, constituted the major barriers, being rated a medium and major obstacle by 56.5% and 48.0% of participants, respectively. The International Continence Society consensus statement^16^ recommended that clinicians “cautiously provide an adequate quantity of reasonable, accurate facts to their patients to enhance the patients’ knowledge” and “improve the patients’ prioritization of PFMT” among the most important tactics to enhance PFMT adherence. The near-universal endorsement of physician recommendation observed in this study, with 91.5% of participants agreeing or strongly agreeing that they would try PFPT if their doctor recommended it, indicates that physician support functions as a strong motivator capable of overcoming the remaining barriers. This is in line with the “credible messengers” model, which aims to alleviate the awareness–action gap for pelvic floor prehabilitation.^17^

In this study, the willingness rate for participation reached 64.0% when PFPT was offered free of charge, substantially higher than the 31% willingness reported for Dutch postpartum women by Moossdorff-Steinhauser et al. However, that study^18^ targeted asymptomatic women for preventive PFMT. In general, the higher willingness observed in the present study may be attributable to the greater symptom burden in this sample (24% reporting UI and 12% reporting prolapse), and Moossdorff-Steinhauser et al similarly found that willingness to participate was higher among symptomatic women.^18^ It should be noted, though, that the expressed willingness may not translate into an equivalently high rate of actual participation. Paquette et al^19^ found that among participants instructed to perform PFMT, real-world effectiveness was only 23%, as 40% of participants did not follow the instructions given. Shannon et al^20^ reported that the completion rate of PFPT was less than 29% among participants from an instructed sample. The disparity between expressed willingness and sustained participation observed elsewhere in the literature should therefore inform the interpretation of the willingness rates found here.

Looking specifically at willingness, the most notable finding was the considerable influence of physician recommendation, which raised willingness from 64.0% to 84.0%—a 20–percentage-point increase that exceeded the effect attributable to removing cost as a condition. This finding is consistent with reports that classes offered by enthusiastic clinicians and regular clinical assessment increase participation in pelvic floor programs.^16^ However, a provider vacuum is noted. Elmahgoub et al^21^ found that only 67.1% of Libyan gynecologists believed physiotherapists were competent to manage pelvic floor problems, a perception that may restrict referral rates and, in turn, limit the real-world reach of the physician effect observed here.

With respect to knowledge, although awareness was reasonably good, 65.5% of participants recognized that pelvic floor exercises may help reduce urinary leakage, and 66.0% recognized that physiotherapy can improve symptoms of POP, specific misconceptions persisted in a minority of participants: 6.5% believed that surgery is the only effective treatment for UI, and 6.0% believed that pelvic floor disorders affect only postmenopausal women. Notably, a substantial proportion of participants (30.5%-47.0% across the 4 knowledge items) remained unsure of the correct answer rather than answering incorrectly, suggesting that uncertainty, more than misinformation, characterizes the principal knowledge gap in this population. This pattern is broadly consistent with the moderate knowledge scores (mean: 8.95/16) reported for Saudi women in Madinah by Bakhsh et al,^9^ who found a significant lack of knowledge regarding the specific types of UI. Koçyiğit et al^22^ similarly reported that structured pelvic floor health education increased knowledge scores from a median of 2.0 to 22.0 (*P* < .001), supporting the view that the knowledge gaps identified here are likely addressable through targeted patient education.

Financial cost was rated a medium or major barrier by 56.5% of respondents, making it the most reported obstacle to utilizing PFPT. Ghandour et al^23^ identified financial concerns as the predominant barrier to seeking care among symptomatic women in Lebanon, regardless of age or education level. Mahfouz et al^24^ similarly found that in more structured healthcare systems, financial barriers were less frequently reported by women, suggesting that this barrier may be policy-related rather than purely individual. Difficulty accessing services was the second most prevalent barrier (medium/major: 48.0%), a finding also echoed by Boulanger et al,^25^ who reported embarrassment as a predominant barrier for women in Jordan, and Salmon et al,^26^ who described the social and emotional distress that women from conservative backgrounds may face when disclosing pelvic health concerns. By comparison, embarrassment specifically about discussing or practicing pelvic floor exercises was rated a medium or major barrier by only 11.0% of respondents, suggesting that in this population, practical barriers of cost and access outweigh social stigma as obstacles to care.

The preference that 72.5% expressed for female physiotherapists is culturally meaningful. Hammad found that Gulf women were less likely to report UI because of the embarrassment they felt in having to consult male doctors.^8^ The preference for technology-based delivery, favored by 39.5% of participants for video- or app-based home programs, aligns with the meta-analysis by Xu et al,^27^ which showed that eHealth interventions have been successful in the prevention of pelvic floor dysfunction (OR = 0.25, 95% CI: 0.14-0.45) and in strengthening self-efficacy (SMD = 2.62). The Cochrane review by Hay-Smith et al^28^ showed moderate-certainty evidence that eHealth delivery was superior to instructions by mail. However, it is still unclear whether remote supervision would be a valid substitute for supervision in the clinic. Family support was reported by 60.5% of participants as increasing their likelihood of participation. This is in line with Terefe et al,^29^ who found that lack of social support was significantly associated with a failure to seek care for POP (AOR = 4.46). A COM-B–based meta-synthesis by Gui et al^15^ identified “family support networks” as a social opportunity that strongly affects pelvic floor exercise behaviors.

In this study population, only 11.0% of participants had ever consulted a physiotherapist for pelvic floor problems and 22.0% had previously tried pelvic floor exercises or physiotherapy in any form, reflecting the generally low utilization of physiotherapy services reported across the Gulf region.^8^ There are many risks and a scarcity of evidence to support the value of pelvic floor exercises practiced without any guidance.^7^ Among people reporting experience, only 6.5 reported improvement, while 27.0% were unsure, again suggesting that most of those people practiced the exercises unsupervised. This is supported by evidence that physiotherapy has more significant impacts when it is practiced in a guided manner.^28^ This gap further supports the description of PFPT having extensive documentation and evidence to support its use but being underutilized. The current study finds no demographic predictors of willingness to receive PFPT, which is somewhat in agreement with the findings of Shannon et al and Aguirre et al,^20,30^ who reported that age, marital status, and education had no bearing on PFPT initiation or adherence.

These findings have practical implications. Almost all respondents (91.5% agreeing or strongly agreeing) were open to trying PFPT if suggested by their physician, which would make routine PFPT counseling a feasible and valuable addition to most OBGYN practices. Financial barriers, reported by more than half of participants, could plausibly be addressed through expanded insurance coverage for physiotherapy services. The knowledge gaps identified, which were characterized more by uncertainty than by firm misconceptions, appear well suited to correction through structured patient education and counseling. Given participants’ strong preference for female physiotherapists, investment in women’s pelvic health services in Saudi Arabia should prioritize the availability of female providers. Finally, the finding that family support increases willingness to participate suggests that family-inclusive educational strategies, such as providing informational materials for spouses or involving family members in initial counseling sessions, may further enhance PFPT uptake.

## Strengths and Limitations

This study’s strengths include the recruitment of the full target sample of 200 Saudi women attending OBGYN clinics and an extensive assessment of knowledge, attitudes, barriers, and willingness across 2 clinically relevant scenarios (PFPT offered free of charge and PFPT recommended by a physician). The bilingual questionnaire, together with adherence to the STROBE guidelines, further supported the study’s methodological rigor. Several limitations remain. Recruitment from a single tertiary center limits external validity, and the findings may not generalize to women in rural regions or to those who are disengaged from the healthcare system altogether. Convenience sampling in clinic waiting areas may also have introduced response bias, since women who agreed to participate may differ systematically from those who declined or from women who do not attend OBGYN clinics at all. The self-administered questionnaire may additionally be subject to social desirability bias, particularly given the culturally sensitive nature of some items. The cross-sectional design limits the ability to measure willingness as an actual behavioral outcome rather than a stated intention; as discussed above, real-world adherence to PFMT recommendations has been reported at only 23%-40% in other populations.^19^ Although the expanded sample permitted inferential analyses of factors associated with willingness, the cross-sectional design remains unable to establish temporal or causal relationships. The identified associations should therefore be interpreted as exploratory rather than predictive or causal, and prospective studies are needed to determine whether the identified factors influence actual PFPT uptake and adherence. Finally, and most importantly, because the questionnaire was a study-specific instrument and not a validated measure, the outcome would have low reliability and therefore weak comparability with other population studies. The need for studies with larger samples, studies that utilize adequate measures such as the Arabic PIKQ, and longitudinal studies that examine the gap between the expressed willingness for PFPT and the actual participation of PFPT is critical to the field.^31^

This research shows that Saudi women attending OBGYN clinics are open to the idea of PFPT. This is especially true when such services are introduced by a physician. This phenomenon was especially true in instances where access to these services was primarily restricted by cost. It was found that a good foundation of beliefs was accompanied by the knowledge gap that was identified during the research. That gap was due to the potential for educational intervention. However, although there is a need for further research to gain insights into the phenomena, the gap between willingness to participate and what was seen in the actual practice should be taken into account when interpreting these findings. Moreover, given the intent of the gap identified, additional studies to validate the findings and measure the effectiveness of interventions in this regard should be carried out.

## Data Availability

The data that support the findings of this study are available on request from the corresponding author.
